# Second-generation anti-amyloid monoclonal antibodies for Alzheimer’s disease: current landscape and future perspectives

**DOI:** 10.1186/s40035-025-00465-w

**Published:** 2025-01-27

**Authors:** Byeong-Hyeon Kim, Sujin Kim, Yunkwon Nam, Yong Ho Park, Seong Min Shin, Minho Moon

**Affiliations:** 1 Department of Biochemistry, College of Medicine, Konyang University, 158, Gwanjeodong-Ro Seo-Gu, Daejeon, 35365 Republic of Korea; 2 Research Institute for Dementia Science, Konyang University, 158, Gwanjeodong-Ro Seo-Gu, Daejeon, 35365 Republic of Korea

**Keywords:** Alzheimer’s disease, Aducanumab, Lecanemab, Donanemab, Gantenerumab, Amyloid-related imaging abnormalities

## Abstract

Alzheimer’s disease (AD) is the most common type of dementia. Monoclonal antibodies (MABs) serve as a promising therapeutic approach for AD by selectively targeting key pathogenic factors, such as amyloid-β (Aβ) peptide, tau protein, and neuroinflammation. Specifically, based on their efficacy in removing Aβ plaques from the brains of patients with AD, the U.S. Food and Drug Administration has approved three anti-amyloid MABs, aducanumab (Aduhelm®), lecanemab (Leqembi®), and donanemab (Kisunla™). Notably, lecanemab received traditional approval after demonstrating clinical benefit, supporting the Aβ cascade hypothesis. These MABs targeting Aβ are categorized based on their affinity to diverse conformational features of Aβ, including monomer, fibril, protofibril, and plaque forms of Aβ as well as pyroglutamate Aβ. First-generation MABs targeting the non-toxic monomeric Aβ, such as solanezumab, bapineuzumab, and crenezumab, failed to demonstrate clinical benefit for AD in clinical trials. In contrast, second-generation MABs, including aducanumab, lecanemab, donanemab, and gantenerumab directed against pathogenic Aβ species and aggregates have shown that reducing Aβ deposition can be an effective strategy to slow cognitive impairment in AD. In this review, we provide a comprehensive overview of the current status, mechanisms, outcomes, and limitations of second-generation MABs for the clinical treatment of AD. Moreover, we discuss the perspectives and future directions of anti-amyloid MABs in the treatment of AD.

## Background

Monoclonal antibodies (MABs) were first developed in 1975 using the hybridoma technology invented by Köhler and Milstein [1], and have been essential in the diagnosis and treatment of debilitating diseases [2–4]. Numerous MABs have been approved by the U.S. Food and Drug Administration (FDA) to treat a variety of diseases, starting with muromonab CD3 (Orthoclone OKT3®), a MAB that inhibits acute rejection in organ transplants [5]. Several MABs for Alzheimer’s disease (AD) that target amyloid-β (Aβ) and tau, as well as inflammation, are currently in clinical trials [6]. Importantly, under the accelerated approval program, the FDA approved anti-amyloid MABs, such as aducanumab (Aduhelm®), lecanemab (Leqembi®), and donanemab (Kisunla™) based on surrogate markers of Aβ plaque removal in the brains of patients with AD [7, 8]. Lecanemab recently became the first disease-modifying drug to transition from accelerated approval to traditional approval, following the FDA’s determination that it has demonstrated clinical benefits [9]. These findings provide valuable evidence upholding the Aβ cascade hypothesis that the aggregation and misfolding of Aβ leads to multiple pathological phenotypes and clinical dysfunctions in AD [10, 11].

Anti-amyloid MABs can be categorized based on affinity, with some showing a preference for binding to monomeric Aβ and others to fibrils. Unfortunately, the first-generation anti-amyloid MABs failed in clinical trials as they demonstrated no significant clinical benefit [12–15]. Notably, solanezumab failed to remove Aβ plaques or inhibit Aβ accumulation in all patients with preclinical AD to moderate AD in four clinical trials [16–18]. These results suggest that targeting monomeric Aβ may not be effective in the treatment of AD. The recently developed second-generation anti-amyloid MABs, such as aducanumab, lecanemab, donanemab, and gantenerumab, target the toxic form of Aβ aggregates (e.g., oligomers, protofibrils, fibrils, and plaques) [19]. These second-generation anti-amyloid MABs have been shown to considerably reduce Aβ deposition, as detected using amyloid positron emission tomography (PET), and slow cognitive decline in clinical trials [20–22].

In this review, we provide a comprehensive overview of recent advances in the clinical trials of four second-generation anti-amyloid MABs: aducanumab, lecanemab, donanemab, and gantenerumab. In addition, we comparatively analyze their mechanisms and limitations. Finally, we explore future directions for innovation and novel technologies required to develop the next generation of anti-amyloid MABs.

## Clinical trials of second-generation anti-amyloid monoclonal antibodies in AD

**Fig. 1:**
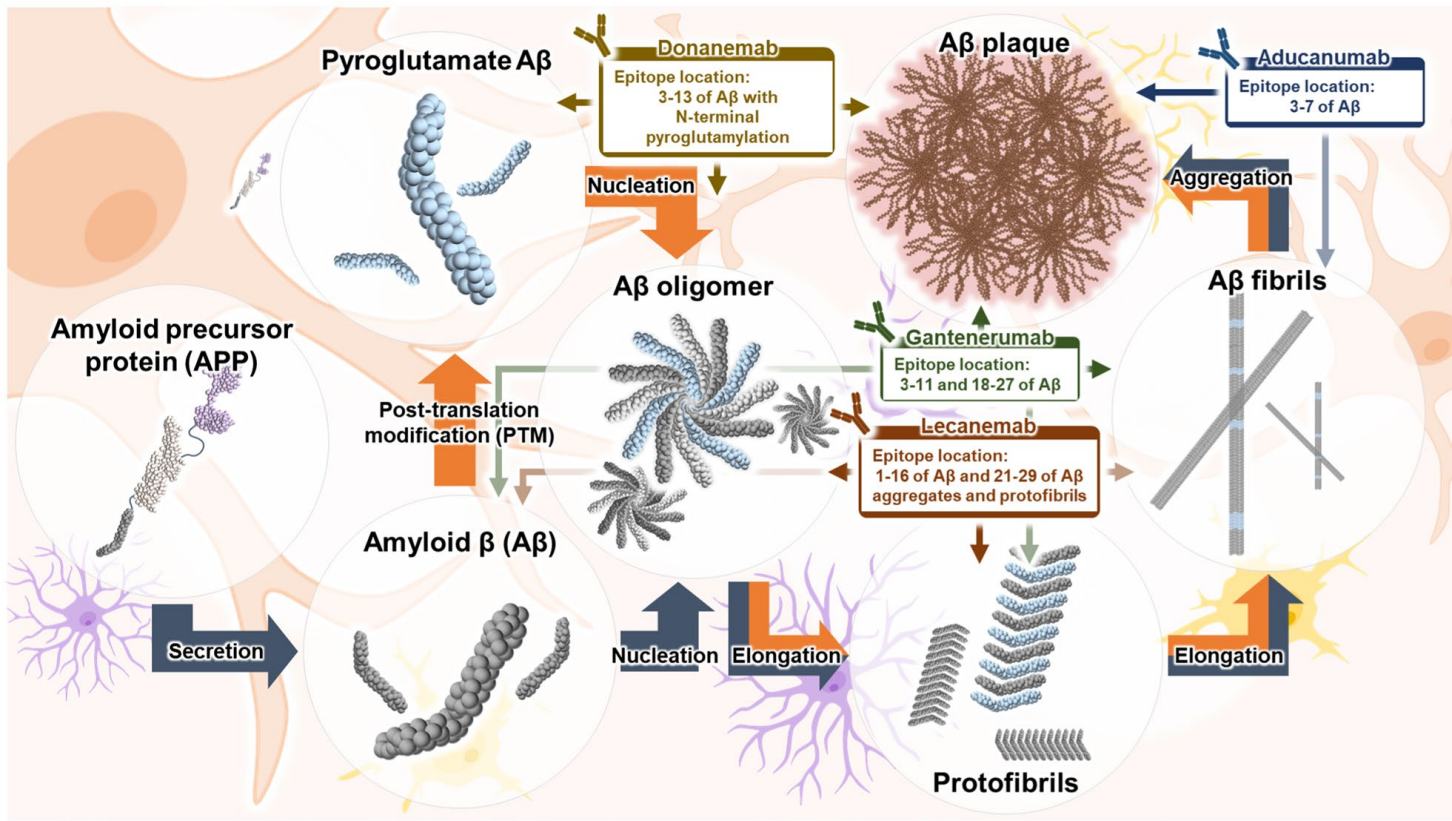
Second-generation anti-amyloid monoclonal antibodies (MABs) target different forms of Aβ. Aducanumab targets Aβ fibrils and plaques. Lecanemab binds to soluble Aβ oligomers, protofibrils, fibrils, and plaques. Gantenerumab targets soluble Aβ oligomers, protofibrils, fibrils and plaques. Donanemab specifically targets the N-terminus of pyroglutamate present in Aβ plaques

Currently, most treatments for AD are symptomatic and do not stop or reverse the progression of AD. Aducanumab, lecanemab, and donanemab are the second-generation anti-amyloid MABs currently approved for clinical use as disease-modifying treatments for AD [21, 23]. Disease-modifying treatments that target Aβ are becoming more popular due to the growing focus on targeting the early stages of disease. In the early stages of AD, disruption of continuous Aβ production and efficient clearance leads to the toxic aggregation of Aβ into misfolded aggregates. Particularly, Aβ aggregates contribute to neurodegeneration and neuroinflammation in the AD brain [24]. Recently, other disease-modifying treatments targeting Aβ, such as gantenerumab, have undergone clinical trials with the aim of treating AD (Fig. 1).

### Clinical trials of aducanumab

Aducanumab was developed from the memory B cells of peripheral blood lymphocytes obtained from healthy elderly individuals without cognitive decline and elderly individuals with cognitive impairment experiencing an unusually gradual decline in cognitive function [25]. Aducanumab mainly targets Aβ plaques by recognizing the amino acids 3–7 of Aβ (Fig. 1) [26]. A single ascending dose study (NCT01397539) of aducanumab, a phase 1 clinical trial, was conducted in 53 patients with mild-to-moderate AD. This study aimed to evaluate the pharmacokinetics, safety, and tolerability of different doses of aducanumab administered via a single intravenous injection in patients with AD [27]. The findings of this clinical trial demonstrated that at 30 mg/kg, patients exhibited excellent tolerance to aducanumab without experiencing severe or serious adverse effects (SAEs), whereas all patients experienced SAEs such as symptomatic amyloid-related imaging abnormalities (ARIA) at a dose of 60 mg/kg [27]. Subsequently, a multiple-dose PRIME study (NCT01677572) of aducanumab, a phase 1 clinical trial, was performed in 197 patients with prodromal-to-mild AD. The study demonstrated that the administration of aducanumab reduced Aβ deposition in the brains of patients with prodromal-to-mild AD in a dose- and time-dependent manner. Moreover, treatment with aducanumab delayed cognitive decline in a dose-dependent manner, as measured by the Clinical Dementia Rating-Sum of Boxes (CDR-SB) and Mini-Mental State Examination (MMSE) scores. However, ARIA-oedema/effusion (ARIA-E) abnormalities were found in groups administered with 1, 3, 6, or 10 mg/kg of aducanumab but not in the placebo group. ARIA-E particularly occurred more frequently in apolipoprotein E (*APOE*) ε4 carriers than in non-carriers [25]. Based on the accumulated clinical results, the ENGAGE (NCT02477800) and EMERGE (NCT02484547) studies established the following clinical criteria: inclusion of patients with Aβ in the brain detected with amyloid PET, inclusion of patients with mild cognitive impairment (MCI), and inclusion of patients who consented to *APOE* genetic analysis (Table 1) [28]. The ENGAGE and EMERGE studies were phase 3 clinical trials in which low (3 or 6 mg/kg) and high (10 mg/kg) doses of aducanumab were administered to 1653 and 1643 patients with MCI due to AD and mild AD, respectively. In the ENGAGE study, aducanumab treatment did not meet the criteria for either primary or secondary clinical endpoints. In contrast, the results of the EMERGE study revealed significant changes in both primary and secondary endpoints in the high-dose aducanumab group; additionally, in the high-dose aducanumab group, the primary and secondary endpoints, such as CDR-SB, MMSE, Alzheimer’s Disease Assessment Scale-Cognitive Subscale (ADAS-Cog) 13, and Alzheimer’s Disease Cooperative Study-Activities of Daily Living Scale for Mild Cognitive Impairment (ADCS-ADL-MCI) scores, decreased by 22%, 18%, 27%, and 40%, respectively. Furthermore, high-dose administration of aducanumab reduced brain Aβ accumulation by 59% and 71% from the baseline in ENGAGE and EMERGE studies, respectively [20]. The difference in results between ENGAGE and EMERGE may be due to the differences in the administration of high-dose aducanumab. In 2017, Biogen modified the protocol to increase the dose of aducanumab to 10 mg/kg for *APOE4*-positive patients. As a result of the protocol change, the number of participants in the high-dose ENGAGE study was smaller than that in the high-dose EMERGE study, and consequently, a smaller proportion of participants received all 14 doses of aducanumab at 10 mg/kg [29]. Specifically, 22% of ENGAGE participants received 14 doses at 10 mg/kg, while 29% of EMERGE participants received 14 doses at 10 mg/kg [30]. Moreover, ENGAGE participants had a shorter average duration of exposure to the 10 mg/kg dose because of the timing of the protocol change. These differences in cumulative exposure to the high-dose aducanumab may have contributed to the differences in efficacy outcomes observed between the two studies. Notably, aducanumab has been successful in slowing cognitive decline and reducing Aβ deposition in patients with AD and has received accelerated approval from the FDA based on the evaluation of surrogate markers for the reduction of amyloid plaques in the brain [8]. However, the most common SAE observed in the ENGAGE and EMERGE studies was ARIA-E, with an incidence of 35.2% in the high-dose aducanumab group [31]. Furthermore, the slowing effects of aducanumab on cognitive decline are still debated [32]. To verify the clinical benefits of aducanumab, the ENVISION study (NCT05310071) recently terminated a phase 3b/4 clinical trial in 1512 patients with MCI due to AD and mild AD.

**Table 1:** Comparison of aducanumab, lecanemab, donanemab, and gantenerumab in phase 3 clinical trials

Anti-amyloid MABs		Aducanumab [20]		Lecanemab [21]	Donanemab [22]	Gantenerumab [53]	
		EMERGE	ENGAGE			GRADUATE I	GRADUATE II
Criteria	AD stage	Early AD	Early AD	Early AD	Early AD	Early AD	
	Key characteristics	50 to 85 years old, Presence of amyloid pathology, Stage 3 and 4 patients as described in the FDA 2018 Guidance, MMSE score: 24–30, CDR global score: 0.5		50 to 90 years old, Presence of amyloid pathology, Objective impairment in episodic memory (WMS-IV LMII)	60 to 85 years old, Presence of amyloid pathology (≥ 37 CL), Presence of tau pathology (> 1.10 SUVr), MMSE score: 20–28	50 to 90 years old, Presence of amyloid pathology, Ratio of phosphorylated tau181 to Aβ_42_ in CSF: 0.024 or higher, Free and Cued Selective Reminding Test (FCSRT): 0.67 or lower, MMSE score: 22–30, CDR global score: 0.5 or 1	
Endpoints	Primary endpoint	CDR-SB		CDR-SB	iADRS	CDR-SB	
		Low dose [Diff vs placebo]: −0.26 (15%), High dose [Diff vs placebo]: −0.39 (22%)	Low dose [Diff vs placebo]: −0.18 (12%), High dose [Diff vs placebo]: −0.03 (2%)	Diff vs placebo: −0.45 (27%)	Low/medium tau population [Diff vs placebo]: −0.68 (36%), Combined population [Diff vs placebo]: −0.67 (29%)	Diff vs placebo: −0.31 (8%)	Diff vs placebo: −0.19 (6%)
	Secondary endpoints	MMSE		ADAS-Cog14	ADCS-iADL	ADAS-Cog13	
		Low dose [Diff vs placebo]: −0.1 (3%), High dose [Diff vs placebo]: −0.6 (18%)	Low dose [Diff vs placebo]: −0.2 (6%), High dose [Diff vs placebo]: −0.1 (3%)	Diff vs placebo: −1.44 (26%)	Low/medium tau population [Diff vs placebo]: 1.83 (40%), Combined population [Diff vs placebo]: 1.70 (28%)	Diff vs placebo: −1.25 (13%)	Diff vs placebo: −1.28 (16%)
		ADAS-Cog13		ADCOMS	ADAS-Cog13	ADCS-ADL	
		Low dose [Diff vs placebo]: −0.70 (14%), High dose [Diff vs placebo]: −1.40 (27%)	Low dose [Diff vs placebo]: −0.58 (11%), High dose [Diff vs placebo]: −0.59 (11%)	Diff vs placebo: −0.05 (24%)	Low/medium tau population [Diff vs placebo]: −1.52 (32%), Combined population [Diff vs placebo]: −1.33 (20%)	Diff vs placebo: 1.11 (9%)	Diff vs placebo: 0.82 (9%)
		ADCS-ADL-MCI		ADCS-ADL-MCI	MMSE	FAQ	
		Low dose [Diff vs placebo]: 0.7 (16%), High dose [Diff vs placebo]: 1.7 (40%)	Low dose [Diff vs placebo]: 0.7 (18%), High dose [Diff vs placebo]: 0.7 (18%)	Diff vs placebo: −2.0 (37%)	Low/medium tau population [Diff vs placebo]: 0.48 (23%), Combined population [Diff vs placebo]: 0.47 (16%)	Diff vs placebo: −0.86 (11%)	Diff vs placebo: −0.86 (13%)
	Biomarker endpoints	Amyloid PET		Amyloid PET	Amyloid PET	Amyloid PET	
		Low dose [Diff vs placebo]: −42 CL, High dose [Diff vs placebo]: −64 CL	Low dose [Diff vs placebo]: −39 CL, High dose [Diff vs placebo]: −54 CL	Diff vs placebo: −59.12 CL	Low/medium tau population [Diff vs placebo]: −88.2 CL, Combined population [Diff vs placebo]: −86.3 CL	Diff vs placebo: −47.5 CL, *Expected result at 84 weeks	Diff vs placebo: −41.5 CL, *Expected result at 84 weeks
				CSF Aβ_42_			
				Increased in lecanemab group			
				Plasma Aβ_42/_Aβ_40_ ratio	Tau PET		
				Increased in lecanemab group	No significant difference	Diff vs placebo: −66.4 CL, * Result at 116 weeks	Diff vs placebo: −56.5 CL, * Result at 116 weeks
				CSF t-tau			
				Decreased in lecanemab group			
				CSF p-tau181	Plasma p-tau271		
		Plasma p-tau181		Decreased in lecanemab group	Decreased in donanemab group	Tau PET	
		Low dose [Diff vs placebo]: NA, High dose [Diff vs placebo]: −0.667	Low dose [Diff vs placebo]: NA, High dose [Diff vs placebo]: −0.777	Plasma p-tau181		No significant difference	
				Decreased in lecanemab group			
				CSF NfL	Plasma neurofilament light		
				Inconsistent results	Inconsistent results		
				Plasma NfL			
				Decreased in lecanemab group			
				CSF neurogranin	Plasma GFAP		
				Increased in lecanemab group	Decreased in donanemab group		
				Plasma GFAP			
				Decreased in lecanemab group			
Adverse effects	ARIA-E	Low dose: 26%, High dose: 35%	Low dose: 26%, High dose: 36%	12.6%, (Symptomatic: 2.8%)	24% (Symptomatic: 6.1%)	23.9% (Symptomatic: 5.2%)	25.8% (Symptomatic: 4.8%)
	ARIA-H	Microhemorrhage		Microhemorrhage	Microhemorrhage	23.7%	22.0%
		Low dose: 10%, High dose: 13%	Low dose: 10%, High dose: 13%	17.3%	26.8%		
		Superficial siderosis		Superficial siderosis	Superficial siderosis		
		Low dose: 10%, High dose: 13%	Low dose: 9%, High dose: 16%	5.6%	15.7%		

CL, centiloid scale; Diff, difference; Early AD means mild cognitive impairment due to AD Mild AD; NA, not available; MMSE, mini-mental state examination; CDR, clinical dementia rating; CDR-SB, clinical dementia rating-sum of boxes; ADAS-Cog13, Alzheimer’s disease assessment scale-cognitive subscale 13; ADCS-ADL-MCI, Alzheimer’s disease cooperative study activities of daily living-mild cognitive impairment scale; PET, positron emission tomography; iADRS, integrated Alzheimer’s disease rating scale; ADCS-iADL, Alzheimer’s disease cooperative study-instrumental activities of daily living; ADAS-Cog14, Alzheimer’s disease assessment scale-cognitive subscale 14; ADCOMS, Alzheimer’s disease composite score; CSF, cerebral spinal fluid; NfL, neurofilament light chain; GFAP, glial fibrillary acidic protein

### Clinical trials of lecanemab

Lecanemab, an MAB that specifically detects the protofibril forms of Aβ, was developed from mouse mAb158 [33]. Lecanemab targets Aβ protofibrils by recognizing amino acids 1–16 of Aβ and 21–29 of Aβ aggregate/protofibrils. Since these epitopes are also exposed in monomers, oligomers, and fibrils, lecanemab binds weakly to them (Fig. 1) [26]. In a preclinical study, short- and long-term administration of mAb158, the murine version of lecanemab (BAN2401), reduced the levels of Aβ protofibrils in the brains and cerebrospinal fluid (CSF) of Tg-ArcSwe mice, a transgenic mouse line expressing the Swedish and Arctic mutant of human amyloid-beta precursor protein (*APP*) gene [34]. A single- and multiple-ascending dose study (NCT01230853) of lecamemab, a phase 1 clinical trial, was performed in 80 patients with mild-to-moderate AD. The phase 1 clinical trial demonstrated the safety and tolerability of lecanemab at sequentially increasing doses, and that it was well-tolerated in all participating treatment groups. SAEs rarely occurred due to drug administration; in particular, the incidence of ARIAs was similar between the lecanemab and placebo groups [35]. Furthermore, Study 201 (NCT01767311), a phase 2 clinical trial, evaluated the safety, tolerability, and efficacy of lecanemab in 856 patients with MCI due to AD or mild AD. In this study, only patients with confirmed evidence of Aβ using PET or CSF testing were eligible for the clinical trial. The primary endpoint for the highest dose of lecanemab administration (twice-monthly, 10 mg/kg) was evaluated through Bayesian analysis of the Alzheimer’s Disease Composite Score (ADCOMS) at 12 months. The calculated probability of showing more than 25% improvement compared with placebo was 64%, falling short of the 80% target. However, the secondary endpoints for the highest dose of lecanemab (twice-monthly 10 mg/kg) demonstrated even greater delay of cognitive deficits at 18 months, with a 30% improvement in ADCOMS, 47% improvement in ADAS-Cog14, and 26% improvement in CDR-SB compared with placebo. Moreover, regarding biomarkers, administration of lecanemab decreased the level of Aβ in the brain and the level of phosphorylated tau (p-tau) in the CSF. The incidence of ARIA-E with lecanemab was 9.9% in patients receiving the highest dose and 14.3% in patients who were *APOE* ε4-carriers [36]. Based on the efficacy of lecanemab in clearing Aβ in the phase 2 clinical trial, the FDA approved lecanemab under the accelerated approval program [7]. A study predicting the long-term treatment with lecanemab using a disease simulation model based on the Study 201 reported that the longer the administration period of lecanemab, the better the clinical outcome [37]. This suggests that early initiation of lecanemab treatment has a potentially greater impact on the progression of AD. Finally, the Clarity AD study (NCT03887455) of lecanemab, a phase 3 clinical trial, was performed in 1906 patients with MCI due to AD and mild AD (Table 1). The primary endpoint, CDR-SB score, showed a 27% delay in clinical decline at 18 months in the lecanemab group compared with the placebo group. The secondary endpoints for lecanemab, including ADAS-Cog14, ADCOMS, and ADCS-ADL-MCI scores, exhibited 26%, 24%, and 37% slowing of cognitive impairment, respectively, in the lecanemab group compared with the placebo group. Furthermore, lecanemab reduced the levels of amyloid burden in the brain by 59.12 (77.92 → 18.8) centiloids (CL) from baseline. In addition, the incidences of ARIA-E and ARIA-haemosiderosis/microhaemorrhages (ARIA-H) in the lecanemab group were 12.6% (Symptomatic ARIA-E: 2.8%) and 17.3% (Symptomatic ARIA-H: 0.7%), respectively [21]. Based on the efficacy of lecanemab in slowing clinical deficits and clearing Aβ deposition in the phase 3 clinical trial, the FDA moved lecanemab to traditional approval [9].

### Clinical trials of donanemab

Donanemab, developed from mouse mE8-IgG2a, recognizes amino acids 3–13 and specifically targets the N-terminal pyroglutamate of Aβ [24, 38]. While donanemab is often characterized as a plaque-specific antibody due to its binding to the N3pE epitope predominantly found in amyloid plaques, it has the potential to interact with various N3pE-containing Aβ species, including soluble, insoluble, and intracellular forms [39, 40]. The interaction between donanemab and various N3pE-containing Aβ species suggests that donanemab has the potential to contribute more comprehensively to Aβ reduction in AD (Fig. 1) [41–43]. Phase 1a (NCT01837641) and 1b (NCT02624778) clinical studies of donanemab have been completed in 100 and 61 patients with mild-to-moderate AD, respectively. Results from the phase 1 clinical trial of donanemab demonstrated that donanemab was well tolerated at both single and multiple doses [44]; however, ARIA was observed in one-quarter of patients receiving donanemab. Single administration of donanemab resulted in changes in amyloid PET only above the dose of 10 mg/kg, and repeated doses of 10 mg/kg and 20 mg/kg resulted in sustained reductions in the deposition of Aβ [44, 45]. Based on these results, the selected dose of donanemab was up to 1400 mg, approximately 20 mg/kg as used in the phase 2 clinical trial. The TRAILBLAZER-ALZ study (NCT03367403) on donanemab, a phase 2 clinical trial, was performed in 272 patients with early symptomatic AD and mild AD. The study only included patients with Aβ and tau depositions as detected using PET. Patients with low (standardized uptake value ratio [SUVR] < 1.10) or high (SUVR > 1.46) tau deposition were excluded from the TRAILBLAZER-ALZ study. Donanemab was intravenously administered to patients at 4-week intervals for 76 weeks (700 mg for the first three administrations and 1400 mg thereafter). The primary and secondary endpoints of donanemab treatment were assessed using the integrated Alzheimer’s Disease Rating Scale (iADRS), CDR-SB, ADAS-Cog13, Alzheimer’s Disease Cooperative Study-Instrumental Activities of Daily Living Inventory (ADCS-iADL), and MMSE scores. The primary outcome showed that there was a significant mean change difference of 3.25 in the iADRS score between the donanemab and the placebo groups. The secondary outcome showed mean changes of − 0.67 and − 0.7 for CDR-SB, 1.83 and 1.70 for ADCS-iADL, and − 1.52 and − 1.33 for ADAS-Cog13 scores in the donanemab and the placebo groups, respectively. These results indicate a tendency toward delayed cognitive decline. Biomarker outcomes for donanemab showed a reduction in amyloid burden by 84.13 (107.6 → 23.47) CL from baseline and a slight slowing of global tau load compared with placebo. Moreover, the group receiving donanemab had a higher incidence of ARIA-E and ARIA-H than the placebo group, whereas neither SEAs nor death was observed in the donanemab group [46, 47]. TRAILBLAZER-EXT (NCT04640077), a follow-up study to the TRAILBLAZER-ALZ study, is currently in progress. Unfortunately, the FDA denied the application for accelerated approval of donanemab based on phase 2 clinical trial data, as it did not meet the minimum requirement of 100 patients to have been treated with donanemab for at least 12 months. Finally, the TRAILBLAZER-ALZ 2 study (NCT04437511) of donanemab, a phase 3 clinical trial, was performed in 1800 patients with early symptomatic AD who had Aβ and tau pathologies (Table 1). The primary endpoint of the study, iADRS, exhibited a 35.1% delay in clinical decline at 72 weeks in the donanemab group compared with the placebo group. The secondary endpoints of donanemab administration, including CDR-SB, ADCS-iADL, ADAS-Cog13, and MMSE scores, also displayed a 37.0%, 39.9%, 32.4%, and 22.9% delayed cognitive decline, respectively, in the donanemab group compared with placebo. The level of amyloid plaques in the brain was reduced by 88.2 CL in the donanemab group compared to that in the placebo group. Notably, 80.1% of the patients treated with donanemab had reduced brain amyloid levels. In addition, the plasma levels of p-tau217 and glial fibrillary acidic protein (GFAP) in the donanemab group decreased by 39.3% and 21.3%, respectively, from baseline. The incidence of ARIA-E and ARIA-H in the donanemab group was 24.0% and 26.8%, respectively [22]. Additionally, the TRAILBLAZER-ALZ 3 study (NCT05026866) on the prophylactic effects of donanemab is currently being conducted in 3300 patients with plasma p-tau217 levels, consistent with early tau pathology, and without cognitive impairment. The TRAILBLAZER-ALZ 4 study (NCT05108922), a phase 3 clinical trial, is comparing the efficacy of donanemab and aducanumab. The TRAILBLAZER-ALZ 5 study (NCT05508789) is currently recruiting patients with early symptomatic AD to evaluate safety and efficacy of donanemab.

### Clinical trials of gantenerumab

Gantenerumab is a novel human anti-Aβ MAB designed using HuCAL® phage display technology to specifically bind to Aβ fibrils [48]. Gantenerumab targets Aβ fibrils by recognizing amino acids 3–11 and 18–27 of Aβ (Fig. 1) [48, 49]. In a preclinical study, intravenous injection of gantenerumab significantly reduced Aβ plaques in the hippocampus, cerebral cortex, and thalamus of PS2APP mice compared to the vehicle group [48]. A multiple-ascending-dose phase I study (NCT00531804) of gantenerumab was performed in 18 patients with mild AD. The patients received 2–7 infusions of intravenous gantenerumab at doses of 60 or 200 mg, or a placebo infusion every 4 weeks. Compared with the placebo group, the level of amyloid plaques in the cortical region was reduced by 15.6% in the group receiving 60 mg of gantenerumab and by 35.7% in the group receiving 200 mg of gantenerumab. Although amyloid plaque levels decreased with gantenerumab administration, two of the six patients in the group receiving 200 mg of gantenerumab developed neuroinflammation or ARIA in the left caudate nucleus and right temporal lobe with the highest level of amyloid reduction [49]. A subcutaneous formulation of gantenerumab (105 or 225 mg) was developed, and the SCarlet RoAD study, a phase 2 clinical trial, comprising 490 participants was initiated. Unfortunately, the SCarlet RoAD study was discontinued early as the target for changes in cognitive delay was not reached [50]. In between the termination of SCarlet RoAD and the initiation of phase 3 clinical trials, a collaborative study called DIAN-TU study (NCT04623242 and NCT01760005) was initiated. The study involved 52 participants with dominantly inherited AD in each treatment group who were assigned to receive either gantenerumab or solanezumab. ARIA-E was observed in 19.2% of patients in the gantenerumab group [17]. Following the termination of the Scarlet RoAD trial, the Marguerite RoAD trial (NCT02051608) evaluating gantenerumab in 389 participants diagnosed with mild AD entered phase 3 clinical testing. However, the Marguerite RoAD trial failed the futility analysis and was converted to an open-label extension (OLE) study. Gantenerumab administered at a high dose of 1200 mg in the SCarlet and Marguerite RoAD OLE studies reduced brain amyloid levels by an average of 59 CL as detected by florbetapir PET. Although one-third of the participants underwent ARIA-E, most were asymptomatic [51]. In addition, patients treated with gantenerumab continued to show reduced amyloid levels. At the end of the three-year SCarlet and Marguerite RoAD OLE study, 80% of the participants treated with gantenerumab showed a reduction in the amyloid burden, resulting in a transition to an amyloid-negative state [52]. Graduate I and II phase 3 clinical trials of gantenerumab efficacy and safety in 1053 and 975 participants with early AD (NCT03444870 and NCT03443973), respectively, failed to demonstrate a significant mean change in cognitive decline (− 0.31 and − 0.19 for CDR-SB from baseline) (Table 1) [53]. Interestingly, the administration of gantenerumab resulted in the partial reduction of amyloid plaques compared to placebo at 116 weeks. Furthermore, gantenerumab treatment resulted in improvements in several soluble biomarkers of AD in the CSF, including reduced levels of total tau, p-tau181, and neurogranin [53]. The observation that gantenerumab reduced amyloid burden but did not improve cognitive function may be related to the characteristics of the patients enrolled in the study. The Graduate I and II clinical trials enrolled patients with MCI due to AD and mild AD who already had significant amyloid plaques in the brain. Moreover, gantenerumab was subcutaneously administered at gradually increasing concentrations, starting at 120 mg for the first 8 weeks, followed by 255 mg until week 20, 510 mg until week 32, and 1020 mg thereafter [53]. Notably, the clinical efficacy of anti-amyloid MABs is expected to be maximized by rapid reductions in brain amyloid levels [54]. Unfortunately, the initial low doses of gantenerumab, 120 and 255 mg through week 20, may have contributed to slower clearance of Aβ, potentially delaying the observation of clinical benefits. Future clinical trials of gantenerumab will need to address the challenges of optimizing dosing strategies and ensuring Aβ clearance to maximize therapeutic efficacy. The phase 3 Graduate I and II studies analyzed a phase 2 study (NCT04592341) evaluating the pharmacodynamic effects of short-term administration of gantenerumab in 192 patients with early AD and phase 3 studies (NCT04339413 and NCT04374253) evaluating the safety of long-term administration of gantenerumab. Furthermore, a new phase 3 clinical trial, the Skyline Study (NCT05256134), evaluating the efficacy and safety of gantenerumab in amyloid-positive, cognitively unimpaired patients at risk of early AD, is currently underway. In addition, a multiple-dose study (NCT01656525) of gantenerumab, a phase 1 trial with 28 participants, and the DIAN-TU study (NCT05552157), a phase 2/3 clinical trial with 220 participants, were discontinued. Future directions for the treatment of AD with MABs currently point to shuttling the transport of MABs across the blood–brain barrier (BBB) to increase target engagement. Trontinemab (NCT04639050), a gantenerumab conjugated to a human transferrin receptor 1 (TfR1)-directed Brainshuttle™ module, demonstrates significantly greater brain uptake than gantenerumab, with brain distribution coefficients reported to be sevenfold to 33-fold higher across various brain regions [55];TfR1-mediated transcytosis is expected to alter the course of immunotherapy for AD.

## A comprehensive analysis of second-generation anti-amyloid MABs in AD

### Comparison of aducanumab, lecanemab, donanemab, and gantenerumab in phase 3 clinical trials

Phase 3 clinical trials of aducanumab, lecanemab, donanemab, and gantenerumab targeted the same cohort of participants, i.e., patients diagnosed with MCI due to AD or mild AD (Table 1). The criteria of AD diagnosis are based on the threshold hypothesis that removal of Aβ before amyloid pathology has reached a certain threshold will inhibit the spread of tau pathology [54]. The “CL” scale was developed to standardize amyloid PET imaging measurements. CL represents that the average value is zero in “high certainty” amyloid-negative patients and an average of 100 in “typical” patients with AD. The ideal range for CL in patients with AD participating in clinical trials of anti-amyloid MABs has been suggested to be typically between 20 and 67 CL [54]. Inclusion criteria for clinical trials of aducanumab were participants aged 50–85 years with (1) an MMSE score between 24 and 30, (2) a CDR global score of 0.5, and (3) disease progression at stages 3 and 4, as described in the 2018 FDA guidelines. In addition, participants between the ages of 50 and 90 with objective episodic memory impairment were included in lecanemab clinical trials. The inclusion criteria for the clinical trials of donanemab were participants aged 60 to 85 years with an MMSE score between 20 and 28, and the presence of tau pathology. Furthermore, participants in the gantenerumab clinical trials were required to be between 50 and 90 years of age, have an MMSE score between 22 and 30, a CDR global score of either 0.5 or 1, a p-tau181/Aβ_42_ ratio in CSF of 0.024 or higher, and a Free and Cued Selective Reminding Test score of 0.67 or lower. Taken together, the criteria for recruiting participants in anti-amyloid MAB clinical trials have become more rigorous, particularly with criteria excluding patients with tau pathology patterns different from those of typical patients with AD as well as those with either too little or too much accumulation.

The therapeutic efficacy of all four anti-amyloid MABs against AD was assessed using CDR-SB scores and amyloid PET scans (Table 1). In clinical trials of anti-amyloid MABs for AD, the changes of CDR-SB scores compared to placebo for aducanumab, lecanemab, donanemab, and gantenerumab were − 0.39 (22%), − 0.45 (27%), − 0.68 (36%) and − 0.31 (8%), respectively. Based on the CDR-SB scores, the order of efficacy of the four anti-amyloid MABs in delaying cognitive decline is as follows: donanemab, lecanemab, aducanumab, and gantenerumab. In clinical trials of anti-amyloid MABs for AD, the changes in amyloid PET values for aducanumab, lecanemab, donanemab, and gantenerumab were − 64 (from 85 to 21) CL, − 55.48 (from 77.92 to 22.44) CL, − 88.0 (from 102.4 to 14.4) CL, and − 47.5 (from 94.44 to 46.9) CL, respectively. Notably, aducanumab, lecanemab and donanemab eliminated Aβ to levels below 20 CL, which is considered amyloid negative. Unfortunately, gantenerumab only reduced Aβ to 46.9 CL and did not reach the amyloid-negative threshold of less than 20 CL. These results from clinical trials suggest that removing Aβ to the negative threshold results in clinical benefit.

The four anti-amyloid MABs have shown different incidences of ARIA in clinical trials involving patients with AD. Aducanumab, lecanemab, donanemab, and gantenerumab showed ARIA-E incidence rates of 36%, 12.6%, 24%, and 25.8%, respectively (Table 1). In clinical trials of anti-amyloid MABs in AD, the Aβ epitope targeted by the antibody and the administered dose could be closely related to the incidence of ARIA-E. In particular, the correlation between the dose/frequency of antibody administration and the occurrence of ARIA is well documented in the clinical trial of lecanemab [36]. The incidence of ARIA increases proportionally with the dosage and frequency of antibody administration. In addition, ARIA occurrence may be related to the Aβ form targeted by anti-amyloid MABs (Table 2). First-generation anti-amyloid MABs mainly targeting Aβ monomers, such as solanezumab and crenezumab, are generally associated with a low incidence of ARIA in patients with AD. However, they showed no clinical benefits in clinical trials of AD [12, 15]. In particular, crenezumab, which significantly binds to multiple forms of Aβ, including monomers, oligomers, fibrils, and plaques [56], showed a lower incidence of ARIA than solanezumab even at higher doses. The primary reason is that crenezumab uses an IgG4 backbone, unlike other anti-amyloid MABs with a different IgG1 backbone [57]. Compared to the IgG1 isotype, the IgG4 isotype weakly binds to FcγR, minimally activates complement, and modulates immune responses. The reduced binding affinity of the IgG4 isotype to FcγR results in decreased FcγR-mediated microglial phagocytic function and leads to anti-inflammatory properties, ultimately contributing to a decreased incidence of ARIA-E. Bapineuzumab, which targets Aβ plaques, showed a high prevalence of ARIA even at low doses and a low frequency of administration [14]. Although clinical studies of bapineuzumab did not detect Aβ deposition in the brain by imaging, the increased concentration of Aβ in plasma suggests that bapineuzumab actively degrades Aβ plaques in the brain. The occurrence of ARIA may be linked to the removal of Aβ plaques deposited within and around blood vessels [58]. In particular, second-generation anti-amyloid MABs that target the oligomeric form and fibrils, such as aducanumab, lecanemab, donanemab, and gantenerumab, have been shown to have a higher incidence of ARIA in the brains of AD patients compared to solanezumab and crenezumab, which target monomeric Aβ [20, 21, 46, 53]. This difference in ARIA incidence appears to be related to the specific Aβ forms targeted by anti-amyloid MABs. In this regard, the impact of anti-amyloid MABs targeting oligomeric Aβ on the incidence of ARIA has drawn substantial research attention. ACU193 is an antibody that specifically binds to Aβ-derived diffusible ligands. ACU193 binds to Aβ oligomers with more than 500-fold selectivity versus Aβ monomers and fibrils [59]. In a phase 1 clinical trial (NCT04931459), ACU193 was administered to 65 patients with MCI due to AD or mild AD. The ARIA-E incidence rate in patients treated with 25 mg/kg ACU193 was 7.1% [60], which was lower than the prevalence observed in patients administered with similar doses of lecanemab, donanemab, gantenerumab, or aducanumab. Therefore, the development of anti-amyloid MABs targeting the oligomeric forms of Aβ may receive increasing attention. However, the clinical efficacy of antibodies that selectively target oligomers, such as ACU-193, has not been confirmed, and further research on antibodies selectively targeting oligomers is needed.

**Table 2:** Association between anti-amyloid MABs and incidence of ARIA

Anti-amyloid MABs	Treatment dosage	Target	ARIA	Clinical benefits	References
Solanezumab	6 mg/kg (Q4W)	Monomeric Aβ	0.9%	No	[12]
Crenezumab	60 mg/kg (Q4W)	Monomeric Aβ (Small extent oligomers)	0.3%	No	[15]
ACU-193	25 mg/kg (Q2W)	Oligomeric Aβ; ADDLs	7.1%	Not available data	[60]
Lecanemab	10 mg/kg (Q2W)	Oligomer / Protofibrils	12.6%	Yes	[21]
Donanemab	20 mg/kg (Q4W)	Pyroglutamate Aβ aggregates	24%	Yes	[22]
Gantenerumab	7 mg/kg (Q2W)	Fibrils/Plaques	25.8%	No	[53]
Aducanumab	10 mg/kg (Q4W)	Plaques	35%	Conflicting	[20]
Bapineuzumab	1 mg/kg (Q13W)	Plaques	11.8%	No	[14]

MABs, monoclonal antibodies; ARIA, amyloid-related imaging abnormalities; ADDLs, Aβ-derived diffusible ligands; Q2W; once every 2 weeks, Q4W; once every 4 weeks, Q13W; once every 13 weeks, ADDLs, Aβ-derived diffusible ligands

### Mechanisms of action of anti-amyloid MABs

**Fig. 2:**
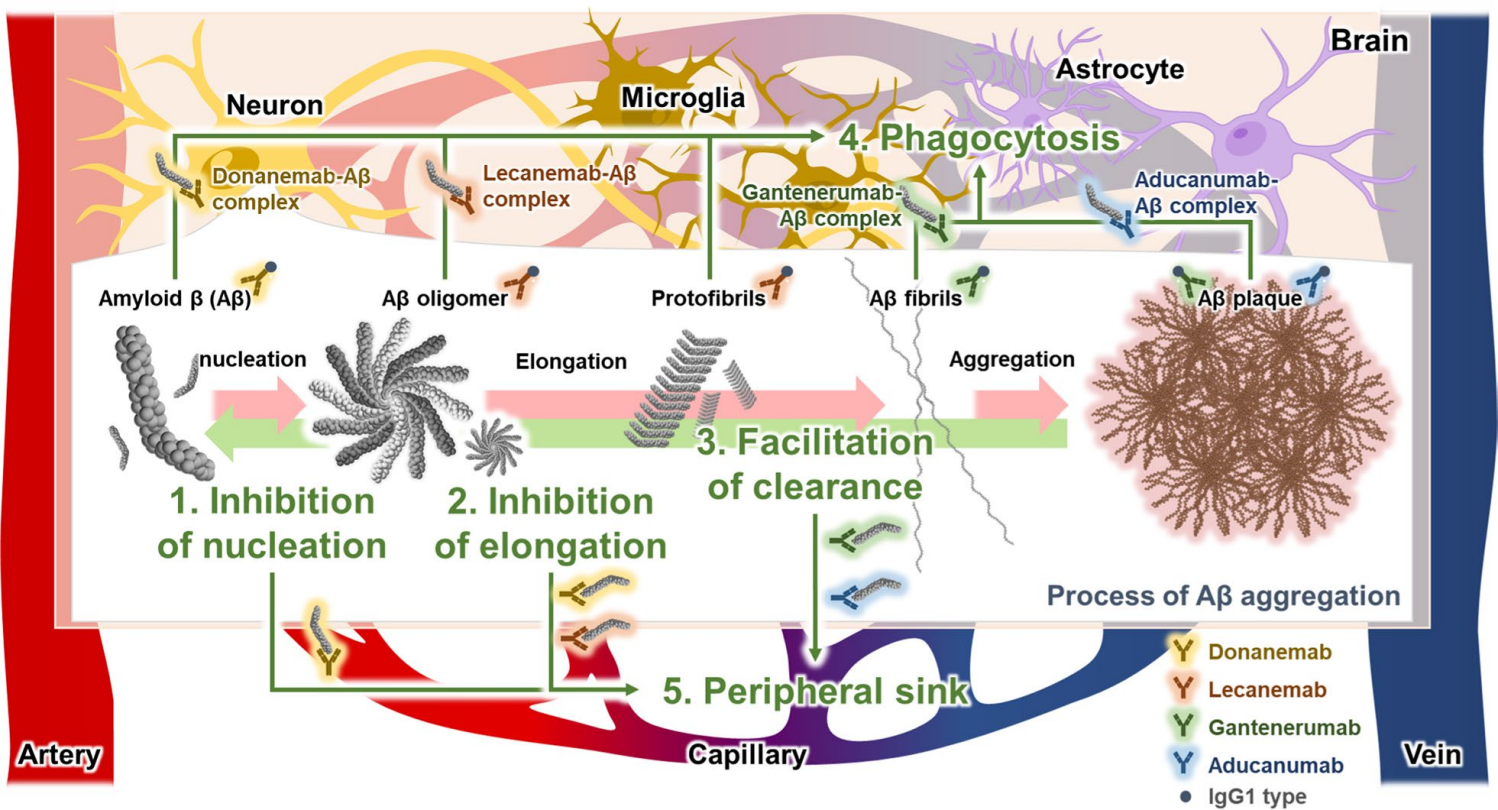
The putative mechanisms by which second-generation anti-amyloid monoclonal antibodies (MABs) reduce the accumulation of Aβ in Alzheimer’s disease (AD). Donanemab-Aβ and lecanemab-Aβ complexes inhibit Aβ accumulation by blocking nucleation and aggregation, and clear Aβ by glial cell-mediated phagocytosis and peripheral sink mechanisms. In addition, gantenerumab-Aβ and aducanumab-Aβ complexes degrade Aβ into sub-aggregates, inducing phagocytosis and peripheral sink mechanisms. Second-generation anti-amyloid MABs have been humanized to the IgG1 subclass

Five putative mechanisms by which anti-amyloid MABs reduce the accumulation of Aβ in individuals with AD have been proposed (Fig. 2) [61, 62]. The first putative mechanism involves binding to Aβ monomers, oligomers, and protofibrils, and interfering with the nucleation of Aβ [25, 48, 63]. Since Aβ oligomers and protofibrils produced by Aβ nucleation are the most toxic aggregates, reducing these forms with anti-amyloid MABs may attenuate the progression of AD [64, 65]. The second mechanism is the blocking of elongation of already-formed Aβ fibrils [66]. Elongation of Aβ fibrils occurs due to the binding of various Aβ forms, such as monomers and dimers, to the endpoints of the fibrils [67]. However, anti-amyloid MABs bind to Aβ fibrils and inhibit elongation by preventing further binding of other Aβ forms to fibril endpoints. The third mechanism is by specifically binding to Aβ aggregates, including fibrils and plaques, and facilitating Aβ degradation to lower-order aggregates. Anti-amyloid MABs break down Aβ into smaller aggregates, prevent reassembly of lower-aggregation-state Aβ, and thus inhibit Aβ-induced neurodegeneration [68]. The fourth putative mechanism is the microglial cell-mediated phagocytosis [61, 62, 69]. Microglia-mediated phagocytosis is induced by microglial recognition of the fragment crystallizable region (FcR) of anti-amyloid MABs bound to Aβ. The four antibodies were humanized with the IgG1 isotype [70], the preferred candidate IgG subclass for therapeutic antibody engineering [71, 72]. The IgG1 isotype is known to act as an effector through the Fcγ receptor [73]. Since all four antibodies have been modified to IgG1 isotypes during the humanization process, differences in phagocytosis based on the IgG1 isotype are not considered to be significant. Interestingly, the efficiency of microglial phagocytosis may be influenced by the species of Aβ. A previous study reported that Aβ fibrils are cleared more efficiently than the oligomeric form of Aβ [74]. These findings suggest that aducanumab and gantenerumab, which target Aβ fibrils, might lead to higher rates of microglial phagocytosis. In other words, microglia target the FcR of anti-amyloid MABs bound to Aβ, removing Aβ from the parenchyma of brains with AD by FcR-mediated microglial phagocytosis. The final putative mechanism is the peripheral sink mechanism, whereby anti-amyloid MABs sequester or dissociate the Aβ in the blood [75, 76]. As a homeostatic mechanism to maintain Aβ levels, the brain with AD effluxes Aβ into the periphery, reducing the formation of Aβ plaques in the parenchyma after anti-amyloid MAB administration.

In summary, anti-amyloid MABs can reduce parenchymal Aβ accumulation and Aβ-induced neuronal death in the brain through five different mechanisms to alleviate Aβ-mediated pathogenesis in AD (Fig. 2): (1) inhibition of Aβ plaque formation; (2) inhibition of Aβ fibril extension; (3) facilitation of Aβ aggregate clearance; (4) microglial-mediated phagocytosis of Aβ; and (5) peripheral clearance of Aβ.

### Limitations of second-generation anti-amyloid MABs

**Fig. 3:**
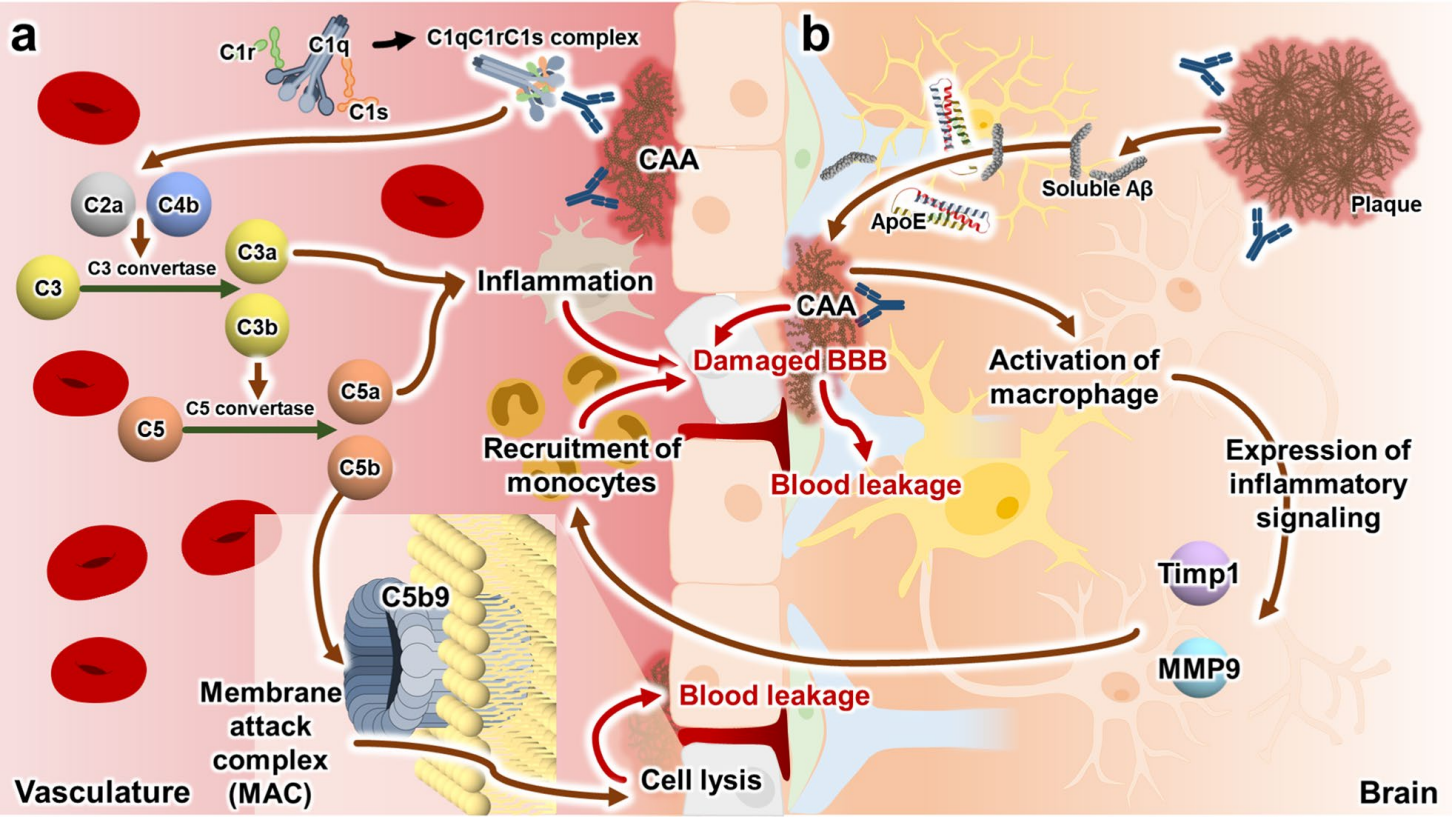
The putative pathophysiological mechanisms leading to the incidence of amyloid-related imaging abnormalities (ARIAs) in the brains of patients with Alzheimer’s disease (AD). **a** The schematic diagram shows the occurrence of ARIA by the classical complement cascade in the vasculature. Anti-amyloid monoclonal antibodies (MABs) induce the formation of C1 complex. The C1 complex disrupts the blood brain barrier (BBB) by forming the membrane attack complex (MAC) C5b-9 through multiple complement signaling pathways. **b** The schematic diagram shows the incidence of ARIA by the FcR-mediated signaling pathways in the brain. During the removal process of Aβ aggregates by antibodies, detritus, such as soluble Aβ, is transported towards the BBB by ApoE. This transported Aβ contributes to the formation of cerebral amyloid angiopathy (CAA). The antibody-mediated clearance of formed CAA not only damages the BBB but also activates macrophages through the FcR-mediated signaling pathway. The activated macrophages induce inflammatory signaling, such as tissue inhibitor of metalloproteinases-1 (TIMP1) and matrix metallopeptidase 9 (MMP9), leading to an increased recruitment of monocytes around the BBB. The BBB is damaged by this inflammatory response

A significant limitation of current antibodies targeting Aβ is the incidence of ARIA. The occurrence of AIRA was first observed in a phase 1 trial of bapineuzumab, one of the first-generation anti-amyloid MABs targeting Aβ plaques [77], and has been reported in the clinical trials of most second-generation anti-amyloid MABs [19]. ARIAs are divided into ARIA-E and ARIA-H based on types of magnetic resonance imaging signal abnormalities. ARIA-E refers to the leakage and accumulation of fluid in the brain, resulting in vasogenic edema or sulcal effusion, which causes hyperintensity abnormalities on imaging scans, such as changes in the cortical folds. ARIA-H refers to an intracerebral hemorrhage resulting in superficial siderosis, observed as hypointense hemosiderin deposition. Almost half of ARIA-E cases are accompanied by ARIA-H, indicating a significant overlap in the pathophysiological mechanisms of these conditions (Fig. 3) [78]. One putative pathophysiological mechanism leading to ARIA involves the formation of the membrane attack complex C5b-9 via the classical complement cascade [79]. Second-generation anti-amyloid MABs circulating in the blood of AD patients first react with accumulated Aβ aggregates in the cerebrovascular wall. This antigen–antibody interaction triggers formation of the C1qC1rC1s complex. The C1qC1rC1s complex activates the complement system via the classical complement pathway, ultimately leading to the formation of the membrane attack complex C5b-9. The membrane attack complex C5b-9 perforates cell membranes, leading to leakage and microhemorrhages. Additionally, anti-amyloid MABs entering the brain cause the dissolution of Aβ plaques, forming soluble Aβ that are transported by ApoE to the vasculature, contributing to the formation of cerebral amyloid angiopathy (CAA) [78]. Anti-amyloid MABs bound to the formed CAA activate perivascular macrophages through FcR-mediated signaling, and these activated macrophages induce the expression of inflammatory signaling molecules, such as Timp1 and matrix metalloproteinase-9 [80]. The recruitment of peripheral monocytes by induced inflammatory signals damages the cerebrovascular wall, resulting in ARIA (Fig. 3b) [81]. This process supports clinical findings that patients carrying the *APOE* ε4 allele have an increased incidence of ARIA, suggesting a genetic predisposition that influences the pathophysiological mechanisms leading to ARIA. Moreover, the presence of the *APOE* ε4 allele exacerbates the process, potentially leading to increased ARIA incidence in patients with AD, as the level of Aβ oligomers in the brains of healthy individuals is approximately 2.7-fold higher than that in the brains of patients with AD and *APOE* ε3/ε3 [82].

It is challenging to safely and effectively deliver peripherally administered anti-amyloid MABs to the brain. When administered intravenously or subcutaneously, only a small fraction of the injected dose crosses the BBB, with only 0.01% to 0.11% of the anti-amyloid MABs present in the plasma known to effectively reach the brain [83–85]. These limitations are important when determining the dose of anti-amyloid MABs in clinical trials for AD. There is a close relationship between amyloid clearance/clinical benefits and the administered doses. Aducanumab, lecanemab, donanemab, and gantenerumab have demonstrated dose-dependent effects on amyloid clearance and clinical benefits. However, high-dose administration of these anti-amyloid MABs also causes side effects, such as an increased prevalence of ARIA [54]. Consequently, these limitations have played a pivotal role in the rejection of both aducanumab and lecanemab by the European Medicines Agency (EMA). Although aducanumab and lecanemab have demonstrated efficacy in reducing Aβ plaques, the relatively modest cognitive benefits were not sufficient to overcome the significant risks associated with ARIA. The EMA’s decision highlights the ongoing challenge of balancing the therapeutic benefits of amyloid clearance with patient safety, especially given the potential for serious adverse events like ARIA-E and ARIA-H.

Several attempts have been made to overcome the limitations of BBB penetration by anti-amyloid MABs. The combination of an antibody recognizing BBB receptors with another antibody targeting Aβ in a bispecific antibody construct, which has two distinct binding domains that can simultaneously bind to different antigens, can enhance BBB penetration [86]. Strategies to facilitate BBB penetration include the construction of bispecific antibodies that enhance TfR1-mediated transcytosis, such as trontinemab, which consists of gantenerumab conjugated to a human TfR1-targeting BBB shuttle fragment. Furthermore, single-chain variable-fragment (scFv) antibodies [87] and nanobodies [88, 89] have superior properties, such as small size, high stability, strong antigen-binding affinity, and high BBB permeability, compared with conventional antibodies. In addition, certain manipulations, such as magnetic or ultrasonic stimulation can temporarily open the BBB in a non-invasive manner to improve the BBB permeability for antibodies [90, 91].

Loss of brain tissue is a critical factor contributing to cognitive dysfunction in AD [92]. Brain atrophy is an objective indicator of disease progression and severity of neurodegeneration. In particular, anti-amyloid MABs, especially second-generation MABs, have been reported to accelerate brain atrophy [20, 36, 46]. It has been suggested that brain atrophy induced by anti-amyloid MABs may be related to the clearance of amyloid plaques from the brains of individuals with AD. However, given that the amount of Aβ peptide present in the AD brain is not abundant [93], it is difficult to induce brain atrophy by clearing amyloid plaques using anti-amyloid MABs. Results from clinical studies on aducanumab, lecanemab, and donanemab in patients with AD indicated that a reduction in plaque volume had minimal impact on changes in global brain volume [94–96]. In addition to clearing amyloid plaques, neuronal loss induced by anti-amyloid MABs results in brain atrophy, which may exacerbate cognitive deterioration in patients with AD. Unfortunately, the molecular mechanisms underlying brain atrophy caused by anti-amyloid MABs, as well as their long-term consequences on AD brain health, remain unknown. Consequently, there are growing concerns on the potential adverse effects of anti-amyloid MABs on cognitive dysfunction and AD progression in patients with AD.

## Perspective and future directions of anti-amyloid MABs: what’s next?

Second-generation anti-amyloid MABs have provided valuable insights that can help resolve the debate surrounding the amyloid hypothesis and improve our understanding of AD pathogenesis and drug development. The following are some of the insights that will be useful for future development and improvement of anti-amyloid MABs for the treatment of AD.

The future direction of next-generation anti-amyloid MABs is to progress toward modifying the structure of antibodies to minimize adverse effects and enhance therapeutic efficacy for the treatment of AD. The prevalent form of antibodies used in recent cancer treatments is a conjugated structure of both antibodies and drugs, and these types of drugs are called antibody–drug conjugates (ADC). An ADC is an immunoconjugate consisting of an antibody conjugated to a payload via a chemical linker [97]. Whether this concept of an ADC can help AD is still on debate. In particular, since small molecules are easier to pass through the BBB than antibodies, the question arises as to why these small molecules should be attached to antibodies. However, these small molecules have limitations, such as low binding to target, short half-lives, off-target effects, and widespread delivery. Antibodies can deliver small molecules more locally and effectively due to their ability to specifically bind to their targets, and they are delivered precisely to the target cell/region, unlike when small molecules are randomly spread and act in the body. This spatial targeting of antibodies can help improve therapeutic effectiveness by delivering small molecules exactly where they are needed, potentially overcoming some of the limitations associated with the broader distribution of such agents within the brain. Moreover, when small molecules are administered alone, they can diffuse or metabolize rapidly in the body. However, such small molecules bound to antibodies can be delivered stably through the antibody and remain in the body for a longer time. Thus, the half-lives of small molecules in the blood are longer, increasing the possibility of reaching target cells. Furthermore, small molecules, when administered alone, can affect normal tissues, potentially leading to increased side effects. However, ADCs can reduce off-target effects by allowing drugs to reach their target. Although antibodies can overcome the limitations of small molecules, in practice, coupling small molecules to antibodies can significantly decrease drug exposure, potentially compromising the therapeutic activity when small molecules already have low target affinity. Hence, several criteria should be considered when selecting a payload in an ADC. These criteria of drugs include solubility, possessing reactive sites for linker conjugation, potency, safety in combination with the linker, and pharmacokinetics after release. Therefore, the concept of ADC, which attaches payloads that reduce the side effects caused by the antibody or maximize the efficacy of the antibody, can help improve the pharmacology of existing antibodies. For example, ADC that combines anti-amyloid MABs with anti-inflammatory corticosteroids (e.g., methylprednisolone and dexamethasone) could effectively reduce the occurrence of ARIA and delay AD progression more than current antibody therapies [19]. ADC allows corticosteroids to target over-activated microglia, which can reduce the development of ARIA by alleviating the inflammatory response caused by activated microglia, without the broader immunosuppressive effects of corticosteroids. Additionally, ADC combining anti-amyloid MABs and Mannitol, which is used to lower increased intracranial pressure, can effectively suppress the occurrence of ARIA based on local efficacy [98, 99]. Moreover, Morphomer® ADC (morADC), a drug candidate unveiled by AC Immune SA at the recent Alzheimer’s Association International Conference, can maximize the efficacy of existing antibodies. The morADC combines monoclonal antibodies and brain-penetrant small molecules developed through the SupraAntigen® and Morphomer® platforms, respectively, to target toxic proteins in neurodegenerative diseases. morADC synergistically increases the aggregation inhibition and clearance of these proteins compared to small molecules or antibodies alone. Recently, a review has proposed the structure of ideal ADC for AD treatment and their possible mechanisms [100]. A possible mechanism for such ADC in AD is as follows: (1) ADC crosses the BBB and accumulates in the brain, (2) the linker of the ADC in the brain is cleaved by extracellular proteases to separate the antibody and payload, and (3) the antibody binds to the antigen and the payload binds to another target, resulting in dual action. Unfortunately, since the efficacy of ADCs based on this mechanism is limited without improved BBB penetration, efforts should focus on the development of antibodies that enhance BBB penetration. In addition, identification of extracellular proteases capable of efficiently cleaving the linker to separate antibodies and payloads in the brain is critical to optimizing this approach.

Targeted protein degradation (TPD) has recently emerged as a promising therapeutic approach for AD. TPD removes protein molecules from inside and outside the cells by engaging in protein degradation pathways [101]. Lysosome‐targeting chimera (LYTAC) is a conjugate that binds to lysosome-shuttling receptors on cell surfaces and to the extracellular domains of target proteins, enabling the targeted degradation of extracellular and membrane proteins. It has been known that LYTAC, produced with an anti-ApoE4 antibody, increases lysosomal uptake of ApoE4, with the potential to treat AD [100]. Moreover, a previous study showed that the attachment of a lysosomal targeting ligand to an antibody induces the antibody to degrade extracellular proteins [102]. Hence, the functionalization of anti-amyloid MABs as LYTACs would allow for the efficient removal of extracellular Aβ aggregates through lysosomes. Proteolysis-targeting chimera (PROTAC), a subtype of TPD, induces the ubiquitination and degradation of target proteins via the ubiquitin–proteasome system [103]. Another specific variation of PROTAC is antibody-based PROTAC (AbTAC). Unlike the traditional PROTACs, AbTACs target membrane proteins. AbTACs utilize bispecific antibodies that bind one of their arms to the target protein and the other to the transmembrane E3 ligase [104]. By modifying anti-amyloid MABs to replace the arm of the antibody that binds to the target protein, AbTAC can also effectively remove intracellular Aβ aggregates through the endosome-lysosome pathway or ubiquitin–proteasome system.

It has been shown that a modification of aducanumab, a second-generation anti-amyloid MAB, provides an effective therapeutic strategy against AD [105]. αAβ-Gas6 is a fusion protein in which the aducanumab scFv that recognizes and binds to Aβ is substituted to the Gla domain of the Gas6 protein. Gas6 is a protein that bridges the interaction between phosphatidylserine and the Tyro3, Axl, and MerTK (TAM) receptors and plays a critical role in mediating efferocytosis by phagocytes [106]. αAβ-Gas6 facilitates the clearance of Aβ through phagocytosis via both microglia and astrocytes, whereas aducanumab exclusively removes Aβ via microglial cells. Unlike aducanumab, αAβ-Gas6 significantly reduces the secretion of pro-inflammatory cytokines in microglia and astrocytes [107]. Furthermore, administration of αAβ-Gas6, compared to aducanumab, reduces the occurrence of CAA and microhemorrhage in AD mouse models [105]. Taken together, the modifications of anti-amyloid MABs are expected to overcome the limitations of existing anti-amyloid MABs and advance their therapeutic efficacy in AD, thereby establishing the next generation of MABs.

With the advancement of anti-amyloid MABs, the identification of biomarkers capable of detecting individuals in the early stages of AD has become crucial for prognostic evaluation. In clinical trials, aducanumab, lecanemab, and donanemab have removed more than 60% of Aβ deposition; however, although the cognitive decline in patients with AD has been slowed, cognitive impairment has not been halted. Furthermore, the levels of neurofilament light chain (NfL), a marker of neurodegeneration, were increased in the CSF and plasma of APP/PS1 mice, although at a slower rate [108, 109]. These findings suggest that while Aβ immunotherapy may be effective against Aβ pathology, it may have a negative effect on other pathological factors. Therefore, it is important to develop biomarkers capable of identifying these other pathological features and to develop therapeutic agents targeting them to optimize the efficacy of Aβ immunotherapy.

Currently, biomarkers commonly used as entry criteria or outcome measures in clinical trials are Aβ and tau [6, 110]. The most common approach for measuring the deposition of Aβ and tau within the brain is using PET scans. Moreover, biomarkers in CSF or plasma, such as the Aβ_42/40_ ratio and the levels of total tau, p-tau181, p-tau217, p-tau231, and microtubule-binding region (MTBR)-tau243, can reflect the pathology of Aβ and tau in the brain [111–113]. In addition to Aβ and tau, there are other biomarkers of AD that are associated with neurodegeneration and inflammation. Biomarkers associated with neurodegeneration in CSF and plasma include NfL, neurogranin, visinin-like protein 1, and synaptosomal-associated protein-25 [114]. Biomarkers related to inflammation, especially gliosis, include GFAP, chitinase-3-like protein 1, and soluble triggering receptor expressed on myeloid cell 2 [114]. The use of both existing and novel biomarkers could enable more precise screening and medication prescription for patients with AD, as well as individuals with MCI due to AD and preclinical AD, who may benefit from early intervention. Given that the annual rate of progression from MCI to dementia is estimated to be 10%–15%, with more than 80% of MCI patients progressing to dementia within six years [115], early diagnosis and intervention are critical to slowing or preventing the progression to AD. Interestingly, circulating RNAs in the blood, especially microRNAs (miRNAs), have shown promise as biomarkers for the diagnosis of preclinical or early stages of AD [116–119]. Several miRNAs have been identified as potential biomarkers for predicting the progression from MCI to AD [120]. Moreover, in preclinical AD, the combination of biomarkers, such as microRNA, p-tau181, NfL, GFAP, and Aβ_42/40_ with amyloid PET or cognitive function tests, including ADAS-Cog13 and MMSE, allows for earlier and more accurate detection of AD [120–122].

Although anti-amyloid MABs have been shown to remove already formed Aβ plaques and ameliorate the toxic events already initiated by these Aβ aggregates, they do not slow plaque formation by inhibiting Aβ production, which is the first step in the amyloid cascade in the pathogenesis of AD. Therefore, combination therapies should be considered, depending on the clinical stage of the disease. First, β-site amyloid precursor protein cleaving enzyme inhibitors and γ-secretase inhibitors/modulators could be considered for patients in the preclinical stage where there is elevated Aβ yet low levels of other tau or neurodegeneration-related biomarkers. This administration can help delay other pathology and the onset of AD by inhibiting excessive Aβ production. Then, when patients enter the prodromal stage due to Aβ accumulation, they are shifted to anti-amyloid MABs to remove the Aβ that has already accumulated. The stage-targeted approach would maximize the benefits of both drugs to create a synergistic effect [123]. In addition, given that Aβ accumulation contributes to tau phosphorylation and neurofibrillary tangle formation [124], combined therapies with drugs that target tau pathologies, such as tau aggregation inhibitors, phosphorylation inhibitors, and antisense oligonucleotides that bind to tau mRNA and block protein expression, may be useful as part of a multi-target combination therapy. Collectively, since AD is a multifactorial disorder involving multiple pathological processes, combination therapies targeting multiple pathological targets are likely to be more effective than single-target therapies once the disease has progressed and various pathologies have been initiated.

## Conclusions

Second-generation anti-amyloid MABs, including aducanumab, lecanemab, donanemab, and gantenerumab, have attracted significant interest in the development of treatments for AD. In clinical trials, anti-amyloid MABs have demonstrated efficacy in reducing Aβ deposition and slowing cognitive decline in patients with AD. However, challenges such as the risk of ARIA, low BBB permeability, low efficacy, and brain atrophy remain unsolved. Our perspectives and future research directions should focus on addressing these challenges. The next generation of anti-amyloid MABs is expected to be developed and introduced through modifications that enhance BBB penetration and therapeutic efficacy while reducing adverse effects. Aligned with these advancements, the identification of biomarkers for the early detection of AD will contribute to increasing the efficacy of anti-amyloid MABs in the treatment of AD. Furthermore, given the complex pathology of AD, drugs targeting other pathologies along with anti-amyloid MABs should be prescribed to patients with AD as a combination therapy.

## Data Availability

Not Applicable.

## Abbreviations

AbTAC: Antibody-based PROTAC
AD: Alzheimer’s disease
ADAS-Cog: Alzheimer’s Disease Assessment Scale-Cognitive Subscale
ADCOMS: Alzheimer’s Disease Composite Score
ADCS-ADL-MCI: Alzheimer’s Disease Cooperative Study-Activities of Daily Living Scale for Mild Cognitive Impairment
ADCS-iADL: Alzheimer’s Disease Cooperative Study–Instrumental Activities of Daily Living Inventory
Aβ: Amyloid-β
ARIA: Amyloid-related imaging abnormalities
ARIA-E: ARIA-oedema/effusion
ARIA-H: Haemosiderosis/microhaemorrhages
MABs: Monoclonal antibodies
ApoE: Apolipoprotein E
BBB: Blood-brain barrier
CDR-SB: Clinical Dementia Rating-Sum of Boxes
CSF: Cerebrospinal fluid
FDA: Food and Drug Administration
FcR: Fragment crystallizable region
iADRS: Integrated Alzheimer’s Disease Rating Scale
MMSE: Mini-Mental State Examination
MCI: Mild cognitive impairment
OLE: Open-label extension
p-tau: Phosphorylated tau
PET: Positron emission tomography
PROTAC: Proteolysis-targeting chimera
SAEs: Serious adverse effects
TfR1: Transferrin receptor 1
